# Gender Differences in Psychological and Behavioral Responses of Infected and Uninfected Health-Care Workers During the Early COVID-19 Outbreak

**DOI:** 10.3389/fpubh.2021.638975

**Published:** 2021-03-11

**Authors:** Qiao Huang, Li-Sha Luo, Yun-Yun Wang, Ying-Hui Jin, Xian-Tao Zeng

**Affiliations:** ^1^Center for Evidence-Based and Translational Medicine, Zhongnan Hospital of Wuhan University, Wuhan, China; ^2^Department of Evidence-Based Medicine and Clinical Epidemiology, Second School of Clinical Medicine, Wuhan University, Wuhan, China

**Keywords:** coronavirus disease 2019, healthcare worker, gender difference, psychological status, protective measures

## Abstract

**Objective:** Understanding gender differences in responses of health-care workers (HCWs) to COVID-19 outbreak is an effective way to promote customized supports.

**Methods:** During February 2020, 103 HCWs infected with COVID-19 (64 females and 39 males) and 535 uninfected HCWs (383 females and 152 males) were recruited in a cross-sectional study. Level of attention, six emotional status, and self-evaluation of eight protective measures were recorded. Multivariable Firth's logistic regressions were applied to explored independent effect of gender.

**Results:** During early outbreak, female HCWs were more likely to give greater attention, adjusted OR:1.92 (95%CI 1.14–3.23) in total HCWs. Higher proportion of anxiety was observed in female HCWs, adjusted OR:3.14 (95%CI 1.98–4.99) for total HCWs, 4.32(95%CI 1.32–14.15) for infected HCWs and 2.97 (1.78, 4.95) for uninfected HCWs. Proportion of pessimism, fear, full of fighting spirit, and optimism were low, and no gender differences were observed. During a later outbreak, a majority of HCWs reported being very familiar with eight protective measures. After training, a proportion of high self-evaluation in hand hygiene, wearing gloves, and surgical masks increased independently in female HCWs, and adjusted ORs were 3.07 (95% CI 1.57–5.99), 2.37 (95% CI 1.26–4.49), and 1.92 (95% CI 1.02–3.62), respectively. Infection status amplified gender difference in anxiety, hand hygiene, and glove wearing.

**Conclusion:** Female HCWs perceived the outbreak seriously, effective emotional and psychological well-ness should be targeted at female HCWs preferentially, and male HCWs should be encouraged to express their feelings and be further trained.

## What Is New?

1. During the early outbreak, female health-care workers (HCWs) gave more attention and presented higher proportion of anxiety.
2. During the later outbreak, higher self-evaluation with hand hygiene, and wearing gloves and surgical mask were observed in both uninfected and infected female HCWs.
3. Infection status amplified gender difference in anxiety, hand hygiene, and glove wearing among HCWs.
4. Effective psychological supports should be targeted at female HCWs preferentially. Male HCWs should be encouraged to express their feelings and be further trained with routine protective measures.

## Introduction

Coronavirus disease 2019 (COVID-19) presented clear evidence of human-to-human transmission and the population is generally susceptible (1). The COVID-19 requires timely diagnosis and effective treatment to prevent the development of severe or critical condition and reduce the risk of death (2). At the early stage of the outbreak, many healthcare workers (HCWs) had to fight against the COVID-19 with limited resources for months. As of March 2020, a total of 3,387 HCWs had been confirmed with COVID-19, and 23 cases had died during their frontline work (3). Uncertainty of COVID-19, colleagues' infections and deaths placed great pressure and threat on frontline HCWs. The necessity of a psychological study on HCWs has been emphasized in many studies (4, 5). Meanwhile, personal hygiene and protection practice in frontline HCWs were emphasized to contain the spread of the outbreak (6).

Generally, sex refers to biological differences between men and women, while gender refers to social roles of males and females (7). Sex- and gender-specific factors were encouraged to be included in medical research as response to the COVID-19 pandemic (8). Even though mechanisms underlying sex- and gender-based effects were still not completely clear, sex-associated biological factors, gender-associated psychological, and behavior responses might interact with COVID-19-related outcomes (9). Men infected with COVID-19 reported a higher susceptibility and mortality (10, 11), sex difference in viral loads, antibody titers, and plasma cytokines were observed (12). Moreover, sex chromosome genes and hormones released promote different responses to viral infections between men and women, such as men have higher levels of angiotensin-converting enzyme 2 (ACE2) coded by the ACE2 gene (13, 14). As COVID-19 outbreak developed, a study on sex differences had attracted increasing attentions. Recently, a large gender difference in COVID-19-related beliefs and behaviors in a global study showed that women were more likely to take the COVID-19 outbreak seriously and to comply with restraining measures (15). To improve effectiveness of gender-based public health policies and achieve gender equity, it is of great importance to take gender-related analyses into preparedness and respond to any outbreak with evidence from past outbreaks (16).

To our knowledge, few studies focused on gender differences in HCWs' psychological and behavioral responses to COVID-19. To close this gap, and to guarantee delivery of optimal interventions on HCWs, we designed this cross-sectional study on both infected HCWs and uninfected HCWs. Differences in psychological burdens and self-evaluation of protective behaviors of male and female HCWs during early COVID-19 epidemic were explored.

## Methods

### Study Design and Participants

From February 22 to February 29, a cross-sectional survey was conducted in Zhongnan Hospital of Wuhan University, where 105 HCWs with confirmed diagnosis with COVID-19 were identified before January 30, 2020, and no new case was infected until now (17). Next, four to six uninfected colleagues were recommended based on close work relationship with infected cases. In few cases, we contacted department leaders to nominate suitable HCWs when no uninfected HCWs were recommended.

This study was a part of a large program and had been reviewed and approved by the Committee for Ethical Affairs of the Zhongnan Hospital of Wuhan University. Electronic consent in an online questionnaire was applied to obtain informed consent from all participants before their enrollment.

### Data Collection

This survey framework was conceptualized according to online interview among frontline HCWs and review of policy documents, guidelines, or consensus for this epidemic. A self-administered electronic questionnaire based on the framework was designed and validated. It presented a high test-retest reliability coefficient of 0.82 (18). Considering the transmission routine of COVID-19 and heavy workload of frontline HCWs, the questionnaire was distributed through WeChat for all eligible participants. To guarantee consistency, tele-interview was held with relevant departments' director for cross-check.

Participant's characteristics were collected, including gender (male/female), age (years), body mass index, marriage status (married/unmarried), job type (doctor/nurse), work capacity (junior/intermediate/associate senior/senior), and experience in treating and nursing in previous epidemic, such as severe acute respiratory syndrome (SARS). Moreover, based on general principles for the management of nosocomial infection control and infection control expert opinion from this project group, the department was categorized into two levels: high risk of nosocomial infection departments (HRDs) and low risk of nosocomial infection departments (LRDs).

Levels of attention and psychologic responses to the epidemic were measured, and a multiple-choice was set for psychological response because of high likelihood of mixed moods during the early epidemic. After the outbreak, training in medical protection was held in this hospital for all frontline HCWs. The protective measures were self-evaluated using a five-point Likert scale, including hand hygiene, wearing gloves, wearing surgical mask, isolation of suspected infectious patients, environmental cleaning and disinfection, wearing goggles or face shield, wearing isolation clothes, and wearing protective clothes. Self-evaluation of protective measures was dichotomized as dependent variables (5 vs. <5).

### Statistical Analysis

Categorical variables were described as frequencies and percentages, continuous variables were shown as mean ± standard deviation (SD), and the Likert scale as median (25–75% percentile). For group comparison between male and female, categorical variables were tested by Chi-square test or Fish's exact test, and continuous variables were tested using Student's *t* test or Wilcoxon's rank-sum test.

To explore independent effects of gender, a series of multivariable binary logistical regression were performed. Since the existence of a rare event, quasi-complete, complete separation, and unstable estimation may appear in logistical regression. Firth's logistic regression, using a penalized likelihood estimation method, was considered as an appropriate method to deal with these potential problems. In addition, each model fit was checked after running logistic regression. For each binary-dependent variable, a logistical regression was performed in uninfected HCWs, infected HCWs, and total HCWs. Interaction between infection group (infected/uninfected) and gender (male/female) was tested using likelihood ratio test, and P for interaction was presented. Each interaction analysis was performed for outcome independently, and multiple correction was not applied.

All analyses were carried out using the SAS software, version 9.4 TS1M6 (SAS Institute Inc, Cary, NC). Two-sided *P* < 0·05 were considered statistically significant.

## Results

Of a total of 105 infected HCWs, 103 cases agreed to participant in this survey; 544 uninfected HCWs were recommended, and 535 cases agreed to participant. Among infected and uninfected HCWs, 64 (62.14%) and 383 (71.59%) were female, respectively.

Basic characteristics were summarized in Table 1. There was no significant difference in age between female and male in infected HCWs, but uninfected males were slightly older than uninfected females. About 70% participants were married; the married rate was significantly low in female uninfected HCWs (61.10%). The type of work varied greatly in gender; the majority of female participants were nurses (71.88% in infected females and 79.90% in uninfected females), while most male participants were doctors (76.92 and 76.97%, respectively). More than half of participants worked in LRDs during this epidemic, and no significant difference between gender was observed. Because average age of recruited participants was <40 years, the rate of experience in treating and nursing in previous epidemic was only about 5%, and the rate in uninfected males was significant and slightly high, 9.87%.

**Table 1:** Basic characteristics between males and females in both infected and uninfected healthcare workers.

**Characteristics**	**Infected HCWs (*****N*** **=** **103)**			**Uninfected HCWs (N** **=** **535)**		
	**Male (*n* = 39)**	**Female (*n* = 64)**	***P***	**Male (*n* = 152)**	**Female (*n* = 383)**	***P***
Age, years, Mean+/-SD	38.62 ± 10.78	36.05 ± 9.96	0.22	36.73 ± 10.00	32.07 ± 8.45	<0.001
BMI, Kg/m^2^, Mean+/-SD	23.67 ± 2.61	21.27 ± 2.55	<0.001	23.67 ± 2.57	20.64 ± 2.43	<0.001
Distribution of BMI						<0.001
<18.5	1 (2.56%)	8 (12.50%)	0.004	4 (2.63%)	84 (22.05%)	
~24	21 (53.85%)	46 (71.88%)		79 (51.97%)	258 (67.72%)	
24~	17 (43.59%)	10 (15.63%)		69 (45.39%)	39 (10.24%)	
Marriage status			0.69			0.001
Married	30 (76.92%)	47 (73.44%)		116 (76.32%)	234 (61.10%)	
Unmarried	9 (23.08%)	17 (26.56%)		36 (23.68%)	149 (38.90%)	
Job type			<0.001			<0.001
Doctor	30 (76.92%)	18 (28.13%)		117 (76.97%)	77 (20.10%)	
Nurse	9 (23.08%)	46 (71.88%)		35 (23.03%)	306 (79.90%)	
Level of department			0.62			0.74
Low risk of nosocomial infection departments (HRDs)	28 (71.79%)	43 (67.19%)		109 (71.71%)	269 (70.23%)	
High risk of nosocomial infection departments (LRDs)	11 (28.21%)	21 (32.81%)		43 (28.29%)	114 (29.77%)	
Work capacity			0.001			<0.001
Senior/associate senior level	19 (48.72%)	9 (14.06%)		54 (35.53%)	28 (7.31%)	
Intermediate level	7 (17.95%)	22 (34.38%)		47 (30.92%)	126 (32.90%)	
Junior level	13 (33.33%)	33 (51.56%)		51 (33.55%)	229 (59.79%)	
Experience in treatment and nursing in other epidemic (e.g., SARS)	2 (5.13%)	3 (4.69%)	1.00	15 (9.87%)	17 (4.44%)	0.017

In infected cases, 14(35.90%) males and 37(57.81%) females stated they paid great attention during early epidemic, *P* = 0.03. But the rate of great attention reached about 85% in uninfected males and females, and there was no statistical difference between genders. For psychological responses to the outbreak, the main performance was normal status (59.24%), followed by anxiety (40.60%), optimistic (32.60) and full of fighting spirit (19.59%), and the rate of both pessimistic and fearful were low. Females showed statistically higher rate of anxiety than males, 39.06% vs. 17.69 in infected HCWs (*P* = 0.03) and 48.30 vs. 27.63% in uninfected HCWs (*P* < 0.001). In both infected and uninfected HCWs, the proportion of fear in females was more than twice that in males, and the difference presented statistical in uninfected group, *P* = 0.02. After the outbreak and training, the median score of self-evaluation was 5 (very familiar) for all eight protective measures in the four groups. In both infected and uninfected HCWs, females reported statistically higher self-evaluated scores than males on hand hygiene, wearing gloves, and wearing surgical masks (Table 2).

**Table 2:** Group comparison between male group and female group on facing conscious and self-evaluation on protective measures to COVID-19.

**When facing the epidemic**	**Infected HCWs (N** **=** **103)**			**Uninfected HCWs (N** **=** **535)**		
	**Male (*n* = 39)**	**Female (*n* = 64)**	***P***	**Male (*n* = 152)**	**Female (*n* = 383)**	***P***
Great attention to the epidemic at early stage	14 (35.90%)	37 (57.81%)	0.03	128 (84.21%)	340 (88.77%)	0.15
**Response to the epidemic at early stage**						
Normal status	27 (69.23%)	39 (60.94%)	0.40	97 (63.82%)	215 (56.14%)	0.10
Anxiety	7 (17.95%)	25 (39.06%)	0.03	42 (27.63%)	185 (48.30%)	<0.001
Pessimistic	2 (5.13%)	4 (6.25%)	1.00	6 (3.95%)	17 (4.44%)	0.80
Fearful	2 (5.13%)	9 (14.06%)	0.27	8 (5.26%)	46 (12.01%)	0.02
Full of fighting spirit	4 (10.26%)	4 (6.25%)	0.72	36 (23.68%)	81 (21.15%)	0.52
Optimistic	9 (23.08%)	12 (18.75%)	0.60	49 (32.24%)	138 (36.03%)	0.41
**Self-evaluation after the outbreak of COVID-19 epidemic**						
Hand hygiene	27(69.23%)	58(90.63%)	0.006	131(86.18%)	357(93.21%)	0.01
Wearing gloves	28(71.79%)	56(87.50%)	0.05	129(84.87%)	355(92.69%)	0.005
Wearing surgical mask	28(71.79%)	57(89.06%)	0.03	131(86.18%)	351(91.64%)	0.06
Isolation of suspected infectious patients	22(56.41%)	46(71.88%)	0.11	108(71.05%)	288(75.20%)	0.32
Environmental cleaning and disinfection	23(58.97%)	49(76.56%)	0.06	117(76.97%)	316(82.51%)	0.14
Wearing goggles or face shield	27(69.23%)	53(82.81%)	0.11	125(82.24%)	337(87.99%)	0.08
Wearing isolation clothes	26(66.67%)	50(78.13%)	0.20	122(80.26%)	330(86.16%)	0.09
Wearing protective clothes	24(61.54%)	45(70.31%)	0.36	115(75.66%)	313(81.72%)	0.11

With adjustment for potential confounders, females were more likely to give great attention to the epidemic in total HCWs (OR: 1.92, [95%CI, 1.14–3.23], *P* = 0.01). In infected and uninfected subgroups, our results showed a 150% increase and 52% increase in rate of great attention in females compared with males. Females were independently associated with increased proportion of anxiety in total HCWs (OR: 3.14, [95%CI, 1.98–4.99], *P* < 0.001), meanwhile, a significant interaction between gender and infection status was observed, *P* < 0.001. It suggested the association differed in infection status (adjusted OR, 4.32 [95%CI, 1.98–4.99] in the infected group; adjusted OR, 2.97 [95% CI, 1.78–4.95] in uninfected group). Other psychologic responses didn't demonstrate significant difference between males and females (Table 3).

**Table 3:** Adjusted effect of gender on facing conscious and self-evaluation on protective measures using Firth's multivariable logistic regressions.

**Gender (Female vs. Male)**	**Odds Ratio (95% Confidence interval)**, *****P***^a^**			**P for interaction**
	**Total**	**Infected HCWs**	**Uninfected HCWs**	
Great attention to the epidemic at early stage	**1.92 (1.14, 3.23), 0.01**	2.50 (0.94, 6.66), 0.07	1.52 (0.78, 2.97), 0.22	0.15
Response to the epidemic at early stage				
Normal status	0.68 (0.44, 1.05), 0.08	0.61 (0.22, 1.69), 0.34	0.72 (0.44, 1.17), 0.18	0.12
Anxiety	**3.14 (1.98, 4.99)**, **<0.001**	**4.32 (1.32, 14.15), 0.02**	**2.97 (1.78, 4.95)**, **<0.001**	**<0.001**
Pessimistic	0.89 (0.33, 2.36), 0.81	1.24 (0.21, 7.19), 0.81	0.81 (0.27, 2.40), 0.71	0.85
Fearful	1.74 (0.81, 3.75), 0.16	3.02 (0.66, 13.78), 0.15	1.35 (0.57, 3.18), 0.50	0.22
Full of fighting spirit	0.64 (0.37, 1.09), 0.10	0.73 (0.16, 3.39), 0.69	0.59 (0.33, 1.06), 0.08	0.09
Optimistic	0.78 (0.49, 1.25), 0.31	0.60 (0.20, 1.81), 0.36	0.78 (0.46, 1.31), 0.35	0.40
Self-evaluation after COVID-19 outbreak				
Hand hygiene	**3.07 (1.57, 5.99), 0.01**	**4.89 (1.20, 19.97), 0.03**	**2.58 (1.19, 5.58), 0.02**	**0.02**
Wearing gloves	**2.37 (1.26, 4.49), 0.01**	3.31 (0.89, 12.31), 0.07	**2.11 (1.01, 4.42), 0.05**	**0.03**
Wearing surgical mask	**1.92 (1.02, 3.62), 0.04**	3.87 (0.99, 15.14), 0.05	1.53 (0.74, 3.18), 0.25	0.20
Isolation of suspected infectious patients	0.97 (0.61, 1.55), 0.90	1.84 (0.68, 4.99), 0.23	0.79 (0.46, 1.36), 0.40	0.54
Environmental cleaning and disinfection	0.99 (0.60, 1.64), 0.96	1.67 (0.59, 4.73), 0.34	0.85 (0.47, 1.54), 0.60	0.54
Wearing goggles or face shield	1.44 (0.81, 2.55), 0.21	2.00 (0.58, 6.85), 0.27	1.32 (0.69, 2.55), 0.40	0.43
Wearing isolation clothes	1.26 (0.72, 2.18), 0.42	1.56 (0.51, 4.75), 0.43	1.16 (0.61, 2.19), 0.66	0.63
Wearing protective clothes	1.13 (0.69, 1.86), 0.63	1.03 (0.37, 2.90), 0.95	1.15 (0.65, 2.05), 0.63	0.80

a *Adjusted for age, BMI level, marriage status, job type, type of department, work capacity, and experience in treatment and nursing in epidemic (e.g., SARS). The bold values mean statistically significant results*.

In self-evaluation of three regular protective measures after the COVID-19 outbreak, a proportion of being fully familiar with hand hygiene, wearing gloves, and wearing surgical masks increased independently in female HCWs, and adjusted ORs were 3.07 [95% CI, 1.57–5.99, *P* = 0.01], 2.37 [95% CI, 1.26–4.49, *P* = 0.01], and 1.92 [95% CI, 1.02–3.62, *P* = 0.04], respectively. Significant interactions of both hand hygiene and wearing gloves with infection status supported the associations and were further strengthened in the infected group. However, self-evaluation of the other five advanced protective measures presented no significant gender difference in the infected group, uninfected group, and total HCWs (Table 3).

## Discussion

Better understanding in emotions in the face of an epidemic and how they differ for female and male HCWs is of great significant to improve HCWs' health during the outbreak. At the early stage of the outbreak, more than half of both female and male HCWs kept a normal status, but females tended to give more attention and presented more anxiety than men. Higher scores in hand hygiene and wearing gloves and surgical masks, which were three recommended and effective measures, were reported in female HCWs. Moreover, the infection status strengthened the gender difference in anxiety and self-evaluation of hand hygiene and glove wearing.

In this study, about 40% participants reported anxiety as response to the early outbreak, which was similar to a previous study (44.6%) using a seven-item Generalized Anxiety Disorder scale (5). There were more females than males at the frontline of the pandemic, and females were more likely to suffer psychological problems (5, 19). The majority was female in our study, and female HCWs had 3.1 times the odds of reporting anxiety compared with male HCWs. A previous study had exhibited higher levels of stress and anxiety in female psychiatric nurses (20). One of negative consequences of increased anxiety was elevated burnout. Recently, many studies on frontline HCWs' burnout during the COVID-19 pandemic were conducted, and a multicenter study with large sample size showed females HCWs presented higher exhaustions score (21), and other two studies reported that the burnout level was higher in frontline female HCWs (22, 23). In addition, an original study and a scoping review showed higher prevalence of stress and depression in female HCWs during the COVID-19 pandemic (24, 25). Female HCWs experienced more psychological problems, which could impact their work performance and health conditions greatly. These findings summarized evidences for preferentially providing timely and high-quality mental care for female HCWs during this ongoing pandemic.

The higher prevalence in female HCWs might result from difference in gender-related work expectations. Female HCWs were expected to spend more time and effort to communicate with patients and deliver mental care to patients in clinical caring work. Strict isolation prevented psychotherapists from entering into isolation wards, and the responsibility of providing psychological care for infected patients was transferred to these HCWs who were under heavy workloads and prolonged working hours. The family–work conflict could be another issue for female HCWs. Frontline HCWs who were female, married, and had children experienced higher levels of anxiety during the COVID-19 pandemic (24). The majority of participants in our study were married, and females were expected to play multiple roles in the family in addition to their career and serve as primary caregivers in the household. Moreover, the closure of schools might require mothers to give their children more patience and energy than usual. A female as the family caregiver and frontline HCW was a gender factor to exacerbate stress exposure (8). Additional expectation greatly increased the likelihood of anxiety in female HCWs.

The sex difference in body structure might bring inconvenience to female HCWs during their clinical work. When caring for infected patients, frontline HCWs wore sealed and heavy protective clothing for about 4–8 h because of shortage of PPE. When female HCWs entered into their menstrual period, the inconvenience of wearing and removing protective clothing to replace sanitary products made them feel powerless and embarrassed. Moreover, the protective clothing impedes their urination, and the mix of urine and blood can increase the risk of infection. In some cases, to avoid the impact of the menstrual period in their work, some had to take progesterone and contraceptives that were associated with increased risk of anxiety (26, 27). So, for female HCWs on their menstrual periods, it should be suggested that they rest instead of work, especially at the initial stage. The previous outbreak indicated that females' needs were largely unmet (28). The demand for feminine hygiene products should attract enough attention, and adult diapers and sanitary pants can be smart alternatives for frontline HCWs.

In our study, the majority of female HCWs (78.30%) were nurses. At the early stage of the outbreak, an influx of patients had overloaded the local medical systems, and nurses were recruited to fight against the outbreak even though they might not be trained with professional and systematic knowledge of infectious disease. In China, family members and paid caregivers played a critical important role in patient care and support because of the shortage of nurse resources (29). However, the contagion of COVID-19 hinderance didn't allow family members and paid caregivers to accompany patients. Overwhelmed workload on these frontline nurses was inevitable (30). During the early stage, limited knowledge about the virus, uncertainty in effective treatment, and risk of infection brought additional challenges to nursing work and made them feel powerless and incompetent in this stressful situation. Moreover, the average age of nurses in this study were about 30 years, which was consistent with the age range of 28,600 recruited nurses across China (31). Less than 5% had experience in treatment and nursing in other epidemics. Being young with a lack of experience made anxiety and fear more common among female HCWs.

Droplet transmission and contact transmission were two main transmission routes of COVID-19. But the shortage of PPE was an indisputable fact in the early stage. In early March, State Council Information Office of the People's Republic of China reported that more than 3,000 HCWs had been infected in hospitals (32). A quality study on doctors stated that doctors gave orders and didn't enter isolation wards when patients were kept stable, while the orders were implemented by nurses for direct contact with patients (33). However, when frontline nurses provided intensive cares and assisted in daily activities for these infected patients in isolation wards, they had to directly contact the infected patients without adequate personal protective equipment (PPE). In daily nursing work, clinical nurses had been trained in hand hygiene and wearing mask; however, studies showed that overall compliances were still low (34, 35). Keeping hand hygiene, wearing gloves, and surgical masks were considered as easy, effective, and convenient measures to prevent COVID-19 cross-infection. These behaviors had been strengthened among nurses because of inevitability of direct contact with confirmed patients and shortage of PPE. Moreover, common and persistent fear and anxiety also contributed to this situation. High attention should be paid to workplace safety, which not only reduces anxiety and fear, but also relieves stress related behaviors.

Lockdown in Wuhan City with a population of over 11 million was considered as an unprecedented and panic event in public health history. During the initial stage of the outbreak, people quarantined in Wuhan experienced a high level of anxiety and fear, and HCWs in Wuhan City were required to face the COVID-19 on the frontline; both fear and anxiety in HCWs may have been further elevated. However, males may have been socialized to keep hidden their anxiety and fear and were less likely to express and discuss troubles and solve emotional problems (36). In this study, both infected and uninfected male HCWs tended to give less attention on the outbreak and have less anxiety and fear. Males have been socialized to think and behave with masculinity. To avoid the feeling of stigma, males are less likely than females to seek mental care, which may lead to high rates of externalizing disorders (36). In this emergency, hospital management should establish appropriate channels to encourage male HCWs to express their feelings and emotions after work. Moreover, the downplay may affect male HCWs' responses to the COVID-19 outbreak, and our study showed that men tended to have lower scores than women in effective personnel protection, including hand washing and wearing masks and gloves. It consisted with conclusions of several studies (37–39). Professional training should emphasize the importance of preventive measures in male HCWs and promote their compliance and practice, including hand hygiene and wearing of masks and gloves.

HCWs' duties to save lives required them to be strong and to persist in the frontline. Multiple support systems, such as separated living space, sufficient PPE, and supportive conversation should be established by the government and hospital management to relieve frontline workers' anxieties (40). Moreover, based on a gender perspective, professional training on self-adjustment skills and psychological reconstruction should be strengthened to protect frontline HCWs.

## Limitations

There are several limitations in our study. First, the principal limitation of our study was an observational study, data were obtained through an online tool, and recall bias and self-reporting problems were inevitable since psychological features and infection status might affect participants' responses to this survey. Second, this study was a part of large survey, and to avoid extra workload, psychological features were not estimated using standard scales with lots of items, but reliability of our tools was acceptable. Third, the difference in age and work capacity between the infected group and uninfected group was found, and heterogeneity in response might be introduced. Even though basic characteristics have been adjusted when exploring potential independent gender differences and interaction effect of infection status on gender difference, residual confounding by the age, work capacity, and other unmeasured factors was still possible. Finally, the generalizability was limited since this is a single center survey.

## Conclusion

This survey was conducted on both infected and uninfected frontline HCWs at the initial stage of the COVID-19 outbreak. Data suggested that female HCWs were more likely to express anxiety and fear and showed higher levels of routine protective measures than male HCWs. To protect our HCWs and to contain the epidemic, it is necessary to understand the gender differences and to promote establishment of customized supports and optimization of hospital management. Effective emotional and psychological comfort should be targeted at female HCWs, and male HCWs should be encouraged to express their feelings and be trained with routine protective measures.

## Data Availability Statement

The raw data supporting the conclusions of this article will be made available by the authors, without undue reservation.

## Ethics Statement

The studies involving human participants were reviewed and approved by the Committee for Ethical Affairs of the Zhongnan Hospital of Wuhan University. The patients/participants provided their written informed consent to participate in this study.

## Author Contributions

Y-HJ and X-TZ had full access to all of the data in the study and take responsibility for the integrity of the data and the accuracy of the data analysis. QH, Y-HJ, L-SL, and Y-YW collected all questionnaire data and processed statistical data. QH drafted the manuscript. L-SL, Y-HJ, and X-TZ made critical revision of the article. All authors agreed with the results and conclusions of this article, and approved the final manuscript.

## Conflict of Interest

The authors declare that the research was conducted in the absence of any commercial or financial relationships that could be construed as a potential conflict of interest.
